# Exosomes: the next frontier in vaccine development and delivery

**DOI:** 10.3389/fimmu.2024.1435426

**Published:** 2024-06-28

**Authors:** Dan Tan, Guangyao Li, Wenyan Fu, Changhai Lei

**Affiliations:** ^1^ Department of Biophysics, College of Basic Medical Sciences, Naval Medical University (Second Military Medical University), Shanghai, China; ^2^ Department of Assisted Reproduction, Shanghai Ninth People’s Hospital, Shanghai Jiao Tong University School of Medicine, Shanghai, China

**Keywords:** exosomes, extracellular vesicles, vaccine, immune responses, antigen presentation

## Abstract

Exosomes are small disk-shaped extracellular vesicles (EVs) that are naturally released into the environment by different types of cells. Exosomes range from 30-150 nm in size and contain complex RNA and proteins. They are widely found in body fluids such as blood, saliva, urine and breast milk and participate in cell communication by functioning as cell messengers. Almost all cell types can transmit information and exchange substances through the production and release of exosomes to regulate proliferation, differentiation, apoptosis, the immune response, inflammation, and other biological functions. Because exosomes exist widely in various body fluids, they are easy to obtain and detect and have the potential for use in disease diagnosis and prognosis detection. Exosomes can be genetically fused with targeted proteins, enhancing their biocompatibility and immunogenicity. Therefore, exosomes are the preferred vector tools for vaccines. In this review, we describe the characteristics of exosomes and discuss their unique and ambiguous functions in the immune microenvironment after infection. In this regard, we explored the ability of exosomes to carry immunogenic virus antigens and to establish adaptive immune responses. Exosomes can provide an interesting platform for antigen presentation and since vaccines are a powerful method for the prevention of infectious diseases, we further review the advantages and disadvantages of the use of exosomes in vaccine preparation. Overall, exosomes are emerging as a promising avenue for vaccine development.

## Introduction

In the ever-evolving landscape of vaccine research and development, a revolutionary approach is gaining significant traction – the utilization of exosomes. These minuscule extracellular vesicles, Which are naturally produced by cells, have emerged as a promising platform for the next generation of vaccines, offering unparalleled opportunities to enhance efficacy, and target delivery, and to personalize immunization strategies against a wide range of infectious diseases, malignancies, and other health challenges (1).

Exosomes are small membrane-bound particles that facilitate the transfer of biomolecules, such as proteins, lipids, and nucleic acids, between cells, playing a crucial role in intercellular communication (2). These tiny cell messengers are secreted by virtually all cell types and have been found to play crucial roles in diverse physiological and pathological processes, including immune regulation, cancer progression, neurodegenerative disorders, and tissue regeneration (3).

## Subsections relevant for the subject

The prospective role of exosomes in vaccine development stems from their unique ability to mimic natural infection processes and interact with the immune system in a targeted and controlled manner (4). Unlike traditional vaccine formulations, which often rely on inactivated or attenuated pathogens or purified antigenic components, exosome-based vaccines have the potential to present antigens in a manner that closely resembles how pathogens interact with the immune system, eliciting a more robust and tailored immune response. Several studies have reported that SARS-CoV-2 receptor-binding domain (RBD) antigen modified human lung globular cell exosomes constructed as a nebulized and inhaled COVID-19 vaccine (RBD-Exosomes vaccine), RBD-Exosomes induced the production of RBD-specific IgA and IgG antibodies against SARS-CoV-2 infection in hamsters, resulting in enhenced mucosal and systemic immunity (5). Moreover, Jiang and Driedonks generated a novel COVID-19 vaccine by modifying the receptor-binding domain (RBD) of the viral S protein on the surface of bacterial-derived external vesicles. In this study, the exosome system was used to characterize the OMV combined with recombinant RBD, and it was determined that the RBD of the novel coronavirus was successfully combined on the OMV. In the development of exosome vaccines, exosomes can be characterized quickly and accurately, and the number of exosomes of different phenotypes can be counted and their proportions can be calculated, which is suitable as a standard detection method for multi-group exosome vaccines. OMV-based exosome vaccines have broad application prospects and can be used as effective means to prevent the spread of COVID-19 and other infectious diseases (6).

One of the key advantages of exosome-based vaccines lies in their versatility as delivery vehicles (7). Exosomes can encapsulate a diverse range of antigenic components, including proteins, peptides, nucleic acids, and even whole pathogen-derived molecules. By loading exosomes with these antigens, researchers can create a unique platform for vaccine delivery that not only enhances the presentation of antigens to the immune system but also offers the potential for targeted delivery to specific cell types or tissues involved in the immune response (8).

Moreover, exosomes offer remarkable opportunities for targeted vaccine delivery (9). By engineering exosomes to display specific surface molecules, researchers can direct them to particular cell types or tissues, such as dendritic cells or lymph nodes, which play crucial roles in initiating and regulating immune responses. This targeted delivery approach could enhance vaccine efficacy while reducing the required dosage and potential side effects associated with systemic administration, thereby improving the overall safety profile of vaccines.

Another prospective application of exosomes in vaccine development is their potential use as adjuvants. Adjuvants are substances added to vaccines to enhance the immune response and improve their effectiveness. Exosomes derived from certain cell types, such as dendritic cells or immune cells, may possess intrinsic adjuvant properties, further boosting the immune system’s response to vaccine antigens. This could lead to the development of more potent and longer-lasting immunity against target pathogens, potentially reducing the need for multiple booster doses and improving compliance with vaccination schedules.

Exosomes also hold immense promise in the field of cancer immunotherapy and the development of personalized cancer vaccines (10). By isolating exosomes from a patient’s tumor cells and loading them with specific tumor-associated antigens or immunomodulatory molecules, researchers aim to create personalized cancer vaccines tailored to each individual’s unique tumor profile. This approach could potentially prime the immune system to recognize and attack tumor-specific targets more effectively, enhancing the efficacy of immunotherapies and improving patient outcomes. Furthermore, exosomes derived from tumor cells may sever as valuable biomarkers that could aid in early disease detection, treatment monitoring, and the development of targeted therapeutic strategies.

Furthermore, exosomes have demonstrated remarkable potential as delivery vehicles for nucleic acid-based vaccines, such as DNA or mRNA vaccines. By encapsulating these genetic materials within exosomes, researchers can protect them from degradation and facilitate their delivery to target cells, potentially enhancing the expression and presentation of antigenic proteins for a more robust immune response. This approach could overcome some of the challenges associated with traditional nucleic acid delivery methods, such as inefficient transfection and potential toxicity, ultimately improving the efficacy and safety of these innovative vaccine platforms.

In addition to their potential in vaccine development, exosomes are also being explored for their prospective role in disease monitoring and diagnostics. The molecular cargo carried by exosomes, including proteins, lipids, and nucleic acids, can serve as valuable biomarkers for various diseases, offering insights into disease progression and treatment response. By analyzing the contents of exosomes isolated from bodily fluids, such as blood, urine, or cerebrospinal fluid, researchers aim to develop minimally invasive diagnostic tests that could facilitate early disease detection and personalized treatment strategies.

## Discussion

Despite the immense potential of exosome-based vaccines, there are still significant challenges to overcome before their widespread clinical application. One of the major hurdles is the large-scale production and purification of exosomes in a consistent and reproducible manner. Researchers are actively exploring various methods, such as cell culture techniques, genetic engineering approaches, and microfluidic devices, to address this challenge and ensure reliable and cost-effective manufacturing processes.

Both pre-clinical and clinical studies show that exosomes play a decisive role in processes like angiogenesis, prognosis, tumor growth metastasis, stromal cell activation, intercellular communication, maintaining cellular and systematic homeostasis, and antigen-specific T- and B-cell responses (11).

Additionally, the precise mechanisms by which exosomes modulate immune responses and interact with different cell types are not yet fully understood. Furthering our knowledge of exosome biology and their interactions with the immune system is crucial for optimizing exosome loading, targeting, and delivery capabilities in vaccine development. Ongoing research efforts are focused on elucidating the molecular mechanisms underlying exosome biogenesis, cargo sorting, and immune modulation, which will inform the design and engineering of exosome-based vaccines (12).

Regulatory and safety considerations also need to be addressed, as exosomes are complex biological entities with the potential for off-target effects or unintended immune responses. Rigorous preclinical and clinical studies will be necessary to evaluate the safety and efficacy of exosome-based vaccines before they can be approved for widespread use. Establishing standardized protocols for exosome characterization, quality control, and safety testing will be essential to ensure the consistent production and reliable performance of these novel vaccine platforms.

Moreover, the successful translation of exosome-based vaccines from research to clinical application will require collaborative efforts among academia, industry, and regulatory authorities. Partnerships between research institutions, biotechnology companies, and pharmaceutical organizations will be crucial in addressing the challenges associated with scalability, manufacturing, and commercialization (13). Regulatory agencies will play a pivotal role in developing guidelines and frameworks for the evaluation and approval of exosome-based vaccines, ensuring their safety and efficacy while facilitating their timely availability to patients.

As the field of exosome research continues to advance, the prospective role of exosomes in vaccine development and delivery is poised to reshape the landscape of immunization strategies, offering innovative solutions for more effective, targeted, and personalized approaches to disease prevention and treatment. The integration of exosome technology with cutting-edge techniques such as synthetic biology, nanotechnology, and bioinformatics could further increase the potential of exosome-based vaccines, leading to transformative advancements in global health and medicine (14).

Compared with traditional virus vaccines, protein-based vaccines and nucleic acid-based vaccines, and exosome-based vaccines as non-cellular vaccines have the advantages of a stable structure, relatively clear composition, high safety, easy preservation, suitability for mass production, and improving the specificity of immune response. Collaborative efforts among researchers, industry partners, and regulatory agencies will be essential in translating the promising potential of exosomes into clinical reality, ultimately improving global health outcomes and contributing to a more resilient and sustainable health care system. As we continue to explore the fascinating world of exosome research, the future of vaccine development and delivery promises to be marked by unprecedented breakthroughs, paving the way for a new era of personalized and targeted immunotherapeutic approaches.

## Data availability statement

The raw data supporting the conclusions of this article will be made available by the authors, without undue reservation.

## Author contributions

DT: Conceptualization, Supervision, Writing – original draft, Formal Analysis, Investigation, Resources, Software, Writing – review & editing. GL: Conceptualization, Data curation, Validation, Writing – review & editing. WF: Funding acquisition, Project administration, Writing – review & editing. CL: Funding acquisition, Project administration, Writing – review & editing.
